# Reduce the application of phosphorus fertilizer in peanut fields and improve its efficiency by using iron modified biochar to adsorb phosphorus recovery products

**DOI:** 10.3389/fpls.2024.1515584

**Published:** 2024-12-17

**Authors:** Junxiao Zhang, Xiangxi Bu, Zhenyu Huang, Changxue Wu, Xiangwen Xie

**Affiliations:** ^1^ Institute of Soil and Fertilizer and Agricultural Sparing Water, Xinjiang Academy of Agricultural Science, Urumqi, China; ^2^ College of Water Conservancy, Shenyang Agricultural University, Shenyang, Liaoning, China; ^3^ Huai’an Water Conservancy Survey Design Institute Co., LTD, Huai’an, China; ^4^ Institute of Water Resources Planning and Development, Jiangxi Academy of Water Science and Engineering, Nanchang, China

**Keywords:** iron-modified biochar, phosphorus fertilizer utilization efficiency, absorption, desorption, yield

## Abstract

**Introduction:**

To address the scarcity of agricultural phosphorus (P) fertilizers and reduce phosphorus accumulation in wastewater, this study employed iron-modified biochar (Fe-B) to adsorb phosphorus from water. The phosphorus-loaded iron-modified biochar (Fe-BP) was subsequently applied to peanut fields. Batch experiments were conducted to determine the optimal adsorption parameters and mechanism of Fe-B for phosphate ions (PO_4_^3−^).

**Methods:**

The field experiment utilized a randomized complete block design, comprising the following treatments: no biochar and no P fertilizer (B0P0), no biochar with conventional phosphate fertilizer (B0P1, CK, P_2_O_5_ at 144 kg ha^−1^), biochar with CK (B1P1), Fe-B with CK (FeB-P1), phosphorus-loaded Fe-B with CK (FeBP-P1), and phosphorus-loaded Fe-B with two-thirds CK (FeBP-P2, P_2_O_5_ at 96 kg ha^-1^).

**Results:**

The results demonstrated that the biochar dosage of 0.05 g (2 g L^-1^) results in a phosphate removal rate exceeding 80%. Optimal adsorption efficiency occurs within a pH range of 6-9, with a sharp decline observed at pH values above 10. The presence of NO_3_^-^, Cl-, and SO_4_^2-^ does not significantly affect the phosphate adsorption capacity of Fe-B, unlike HCO_3_^-^ and CO_3_^2-^, which reduce it. After the fifth desorption and recycling process, the adsorption capacity of the biochar decreased to 24%. The peanut yield in the FeB-P1 treatment was 50.8% higher than that in the FeBP-P2 treatment. While the phosphorus recovery efficiency (REP) does not significantly differ between FeBP-P2 and B1P1 treatments, both are superior to B0P1. Moreover, FeBP-P2 facilitated the available phosphorus concentration in the root zone.

**Discussion:**

Overall, phosphorus-loaded iron-modified biochar reduced the required amount of phosphorus fertilizer, maintain peanut yield, and enhanced phosphorus fertilizer utilization efficiency.

## 1 Introduction

Phosphorus is essential for crop growth in agriculture, yet 30–40% of the world’s arable soils have low phosphorus levels (Zhu et al., 2018). Since the 1950s, the demand for phosphate fertilizer has increased to ensure food security for the growing global population (Alewell et al., 2020). The FAO reported that global phosphate fertilizer consumption reached 47.4 million tons in 2015, with an annual growth rate of 2%., increasing to 49.1 million tons by 2022. However, high-quality phosphate reserves are expected to be exhausted within 50 to 400 years (Zou et al., 2022). Excessive phosphorus application, particularly when crops do not cover the soil, heightens the risk of phosphorus loss through leaching, runoff, and erosion (Schoumans et al., 2014). Loss rates of phosphate fertilizers in natural environments can reportedly reach as high as 80–90% (Dimkpa et al., 2020). The primary cause of water eutrophication, phosphorus leaching into deep soil layers, leads to widespread ecological damage, exacerbating environmental pollution, challenging global sustainability, and causing significant financial losses (Sharpley et al., 1994; Hou et al., 2020). Additionally, excessive chemical fertilizer use has degraded cultivated land quality, resulting in soil salinization and organic matter depletion (Tripathi et al., 2020). In this context, slow-release phosphate fertilizers offer a solution by improving phosphorus utilization efficiency (Li J. et al., 2022). These fertilizers extend nutrient availability for plant uptake, reducing environmental nutrient losses (Bindraban et al., 2015). Marcińczyk and Oleszczuk (2022) suggest that biochar may be a promising alternative material for producing slow-release phosphate fertilizers.

Biochar, a stable material derived from biomass pyrolysis, possesses high carbon content, a large surface area, porosity, and abundant functional groups (Tomczyk et al., 2020). Widely utilized in soil improvement (Qian et al., 2023), sewage treatment (Gonzalez et al., 2021), carbon sequestration (Yang et al., 2021), and emission reduction (Lehmann et al., 2021). Meanwhile, its porous structure enables phosphorus adsorption, with reported phosphate adsorption capacities of 4 mg P g-1 for biochar derived from sugarcane and miscanthus (Trazzi et al., 2016). Despite this, the phosphorus content of biochar remains significantly lower than that of mineral fertilizers such as commercial superphosphate (>46% P2O5). Moreover, traditional pyrolysis-produced biochar exhibits limited surface functional groups with predominantly negative charge, reducing phosphate adsorption efficiency (Chintala et al., 2016). Metal-based materials, particularly iron, exhibit high selective adsorption capacities for phosphorus due to their abundance and small solubility product constant (Ksp) of metal phosphates (Bao et al., 2024). Dong et al. (2016) and Wen et al. (2021) found in their research that iron modified biochar can reduce the impact of harmful substances on plants, and thus increase dry matter accumulation. Phosphorus adsorbed on biochar can generally undergo slow release via an adsorption-desorption equilibrium (Chen et al., 2017). However, the direction of phosphorus deposited on biochar is uncertain, as it may dissolve and face similar soil constraints as conventional mineral phosphate fertilizers (Bacelo et al., 2020). Additionally, application of iron-modified biochar to farmland may reverse adsorbing soil phosphorus due to its numerous adsorption sites (Wu et al., 2020). Thus, investigating the adsorption and slow-release mechanisms of iron-modified biochar phosphate, along with its impact on available phosphorus in farmland, holds significance for sustainable phosphate fertilizer development.

This study aimed to investigate the effects of phosphorus-loaded iron-modified biochar slow-release fertilizer on soil available phosphorus. Two hypotheses were tested in this work. First, we explored the adsorption mechanism of iron modified biochar on phosphate, and second, we explored the effect of iron modified biochar loaded with phosphorus as a phosphorus slow-release fertilizer on the spatial distribution of available phosphorus in farmland soil.

## 2 Materials and methods

### 2.1 Preparation of Fe-B and P-loaded Fe-B

The biochar was derived from maize straw (Northeast China) pyrolyzed at 600°C for 2 h. The collected biochar was washed by aqua pura to remove the ash, dried, and crushed through an 80 mesh sieve. For large-scale application and cost-saving, the alkaline impregnation method was used for this experiment. To prepare the Fe-B, a part of the maize straw biochar was immersed in 1 M FeCl_3_ at a 1:12.5 (w/v) ratio (Fe:C is 0.7), and stirred vigorously for 2 h followed by the dropwise addition of NaOH (pH 10-11) to adjust pH to 11. Seal and oscillate for 24 h. Rinse with deionized water until neutral. The FeB were finally dried to a constant weight at 50°C (Wang et al., 2019).

### 2.2 Adsorption experiment of Fe-B

P adsorption capacity were calculated by Equation 1:


(1) $$q_{e}=\frac{(C_{0}-C_{e})V}{m}$$

where $q_{e}$ is the phosphorus adsorption capacity of Fe-B at equilibrium, mg·g^-1^; *V* is the volume of the solution, L; *m* is the mass of Fe-B, g; $C_{0}$ and $C_{e}$ are the initial and equilibrium concentrations of phosphorus in solution, mg·L^-1^.

#### 2.2.1 Adsorption kinetics

For kinetics adsorption experiments, 0.05 g of Fe-B samples were mixed with 50 mL of phosphate solution and shaken at 170 ± 5 r/min at 25 ± 1°C. The supernatant was collected at specific times(5min, 10min, 15min, 20min, 30min, 60min, 120min, 240min, 480min, 720min, 1440min, and 2880min)using a 0.45 μm millipore filter. Finally, the P adsorption kinetics of the biochar samples were fitted by two classical models: the pseudo-first-order kinetics Equation 2 and the pseudo-second-order kinetics Equation 3.


(2) $$q_{t}=q_{e}\;(1-e^{-k_{1}t})$$

(3) $$q_{t}=\frac{q_{e}2k_{2}t}{1+q_{e}k_{2}t}$$

Where $q_{e}$ is the adsorption capacity of Fe-B at equilibrium, mg·g^-1^; $q_{t}$ is the adsorption capacity of the adsorbent at time t, mg·g^-1^; $\;k_{1}$ and $k_{2}$ are adsorption rate constants.

#### 2.2.2 Adsorption isotherm

For adsorption isotherm experiments, 0.05 g of Fe-B samples were mixed with 50 mL of phosphate solution and shaken at 170 ± 5 r/min at 25 ± 1°C. The solution concentrations are 1, 5, 7, 10, 15, 17, 23, 25, and 30 mg·L^-1^. Finally, the P adsorption isotherm of the Fe-B samples were fitted by two classical models: the Langmuir model Equation 4 and the Freundlich model Equation 5.

Langmuir model:


(4) $$q_{e}=\frac{k_{l}q_{m}C_{e}}{1+k_{l}C_{e}}$$

Freundlich model:


(5) $$q_{e}=k_{f}C_{e}^{-n}$$

where $q_{e}$ is the adsorption amount of the adsorbent at the equilibrium time, mg·g^-1^; $q_{m}$ is the saturated adsorption capacity, mg·g^-1^; $C_{e}$ is the solution concentration at equilibrium, mg·g^-1^; $\;k_{l}$ and $k_{f}$ are adsorption equilibrium constants; *n* is the strength constant.

### 2.3 Field experiment analysis of P-loaded Fe-B

#### 2.3.1 Field layout and treatments application

Field experiments were carried out at the Beidianzi Experimental Station (121°47′E, 42°01′N) located in the Liaoning Province of Northeast China during the growing seasons of 2021 and 2022, spanning from May to September. The study area is situated in a north-temperate zone with a semiarid continental monsoon climate. The region experiences an annual average air temperature of 6.1°C, with an average evaporation of 1780.5 mm and an average annual precipitation of 307 mm. Precipitation and temperature data for the two years were acquired from a local weather station, as depicted in **Supplementary Figure S1**. The soil texture was showed in **Supplementary Table S1**.

The experiment was a randomized complete block design comprising six treatments combinations over three replicates included no biochar and no phosphorus fertilizer (B0P0), no biochar and conventional phosphate fertilizer (B0P1, CK, P_2_O_5_ is 144 kg·ha^-1^), biochar and conventional phosphate fertilizer (B1P1), iron modified biochar (Fe-B) and conventional phosphate fertilizer (FeB-P1), phosphorus-loaded Fe-B and conventional phosphate fertilizer (FeBP-P1), phosphorus-loaded Fe-B and two third conventional phosphate fertilizer (FeBP-P2, P_2_O_5_ is 96 kg·ha^-1^). When preparing a large quantity of phosphorus-loaded iron-modified biochar (Fe-BP), with a phosphorus concentration set at 5 mg/L, phosphoric acid ions in the solution undergo multiple absorption cycles by Fe-B through artificial stirring. After reaching adsorption saturation (2h) of Fe-BP, followed by natural air drying, the Fe-BP is ready for field experiment. The amount of biochar is 24 t·ha^-1^ (1% of the soil mass).The biochar was fully mixed with the upper 15cm soil layer by rotary before sowing.

The test peanut was the cultivar *Baisha*, which is widely planted in the study area. The plant spacing was 15 cm and planting depth of 5 cm with 2 seeds per hole. Each plot was 6.67 m^2^. The planting density was 180,000 hills hm^-2^. Based on the traditional fertilization method in the experimental station. N was applied as urea (156 kg· ha^-1^ N). K was applied as potassium sulfate (144 kg· ha^-1^ K_2_O). P was applied superphosphate. The field was irrigated up to 90% of the water content at field capacity (FC) when the soil moisture content dropped to 50-60% FC. Other managements were in line with local farmer practices to avoid yield losses.

#### 2.3.2 Soil available phosphorus

Soil drilling method was used on the 30th, 74th and 110th days after fertilization. Soil samples were collected at 20 cm intervals within 0 ~ 60 cm of 0 cm (right below the drip irrigation belt), 17.5cm (peanut side), and 25cm (ridge side). After the collected soil samples were air-dried in a ventilated place in the room, they were crushed through a 2mm sieve, and 2.50g of air-dried soil samples were weighed and extracted with 0.5mol·L^-1^ NaHCO_3_ solution. The phosphorus concentration of the extracted solution was determined by ultraviolet spectrophotometer (2700) produced in Shimadzu, Japan. Soil available phosphorus is calculated according to Equation 6:


(6) $$p=\frac{\rho \times V\times ts}{m\times k}$$

Where: *P* is soil available phosphorus content, mg·kg^-1^; *ρ* is the mass concentration of P in the measured curve, mg·L^-1^; *V* is the constant volume at the time of color development, mL; *ts* is the extraction multiple (the ratio of the total volume of the extract to the volume of the extracted liquid during color development); *m* is the quality of air-dried material, g; *k* is the coefficient of mass of air-dried soil replaced by dried soil.

#### 2.3.3 Peanut yield and phosphorus fertilizer utilization efficiency

The center of each plot (1 m^2^) was harvested for yield determination, and yield was determined after air-drying (standardized to 14% water content) (Zhang et al., 2021). Two indicators of P fertilizer use efficiency were calculated as follows:


(7) Recovery efficiency of P, REP, %= (PA−P0A)/Padded×100%

(8) Agronomic efficiency of P, AEP, kg/kg = (PY−P0Y)/Padded

### 2.4 Biochar characteristic

The crystallinities of Fe-B were determined by X-ray powder diffractometry (XRD) (Bruker, D8, Advance, Germany). The shape and size of the samples were analyzed using scanning electron microscopy (SEM) (Hitachi Regulus8230, Japan). Fourier transform infrared spectrometry (FTIR) spectra were conducted to identify the surface functional groups by a Nicolet Avatar 370DTGS spectrophotometer (IR Tracer 100, Japan).

### 2.5 Statistical analysis

Data analysis were computed by using Origin 2023 (Origin Lab, Northampton, MA, USA). Differences between the treatments were analyzed through the one-way ANOVA followed by the LSD test (0.05level).

## 3 Results and discussion

### 3.1 Fe-B adsorption performance

**Figure 1:**
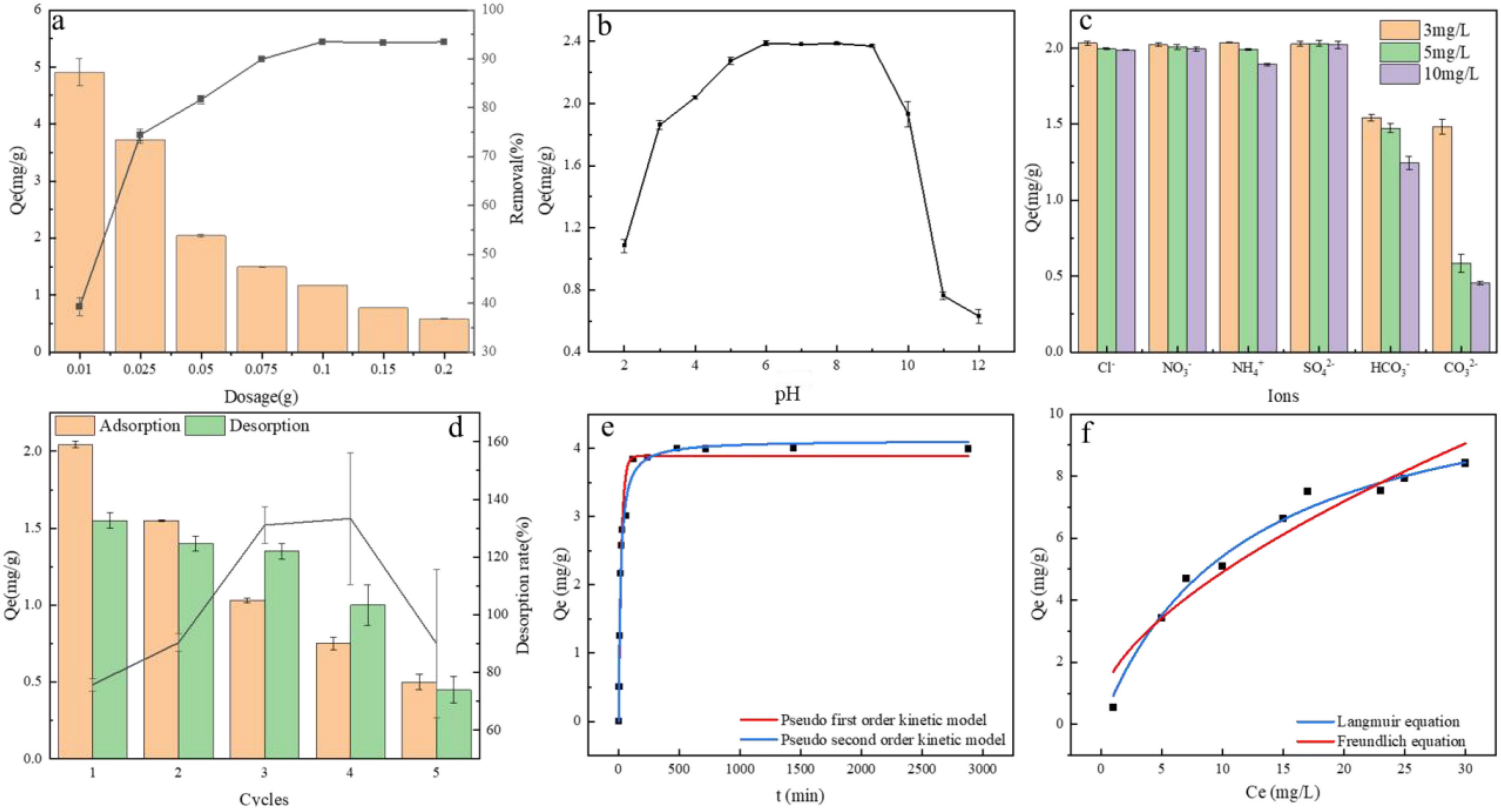
**(A)** Effect of adsorption dosage on adsorption capacity and removal rate; **(B)** Effect of initial solution pH on adsorption capacity; **(C)** Effect of coexisting ions on adsorption capacity; **(D)** Desorption and recycling; **(E)** Kinetic fitting curve for phosphate adsorption by FeB; **(F)** Fitting of Langmuir and Freundlich isotherm adsorption models for Fe-B. Fe-B, iron modified biochar; PO_4_^3−^, phosphate ions.

With increasing Fe-B concentration, phosphate removal efficiency correspondingly improves (**Figure 1A**). This enhancement is due to the more adsorption sites, which enhances the contact area between phosphate and these sites (Li M. et al., 2016). However, once the adsorption sites reach saturation with further Fe-B addition, the removal efficiency remains unchanged as no additional active sites remain for adsorption (Yang et al., 2018). At an Fe-B concentration of 0.05 g (2 g L^-1^), the phosphate removal rate exceeds 80%.

When the solution pH is between 5 and 8, Fe-B adsorbs 2.4 mg·g^-1^ of phosphate, as depicted in **Figure 1B**. As the pH increases from 8 to 10, the adsorption slightly decreases, yet Fe-B retains a high adsorption capacity over this broad pH range, which is crucial for practical applications (Xu et al., 2021). Phosphate exists in four forms: H_3_PO_4_, H_2_PO_4_^-^, HPO_4_^2-^, and PO_4_^3-^ (Iheagwara et al., 2013). At a pH below 2, phosphate predominantly exists as H_3_PO_4_, which is challenging to adsorb, resulting in a low adsorption capacity for Fe-B (Li R. et al., 2016). As the pH increases to 2-4, H_3_PO_4_ diminishes, and H_2_PO_4_^-^ becomes more prevalent, leading to a gradual rise in Fe-B adsorption. In the pH range of 4-6, H_2_PO_4_^-^ is the dominant form, and the hydroxyl groups on Fe-B can undergo ligand exchange with H_2_PO_4_^-^, thus maintaining a high phosphate adsorption levels. When the pH ranges from 6 to 10, H_2_PO_4_^-^ gradually decreases, while HPO_4_^2-^ increases; ligand exchange continues to support Fe-B’s high phosphate adsorption (Du et al., 2022). However, as the pH exceeds 10, the OH^-^ concentration significantly, enhancing electrostatic repulsion. This repulsion, combined with competition between OH^-^ and phosphate for adsorption sites, reduces the phosphate adsorption efficiency of Fe-B rapidly when the pH is between 9 and 12 (Wendling et al., 2013).

The enhanced phosphate adsorption capacity of Fe-B primarily depends on its positively charged surface-active sites. In actual wastewater, high concentrations of various anions often compete with phosphate for these sites (Lin et al., 2021). Common interfering ions in wastewater include NH_4_^+^, NO_3_^-^, Cl^-^, SO_4_^2-^, HCO_3_^-^, and CO3^2-^. This study found that NO_3_^-^, Cl^-^, and SO_4_^2-^ had minimal impact on Fe-B’s adsorption capacity (**Figure 1C**). Increasing anion content can trigger ion competition and enhance electrostatic repulsion, reducing phosphate adsorption. However, the primary mechanism for phosphate adsorption by Fe-B involves complex formation through coordination between surface-loaded iron oxides and phosphate ions, which makes it less susceptible to interference from coexisting ions (Zhang et al., 2020; Huang et al., 2020). NH_4_^+^ slightly reduced phosphate adsorption efficiency, while HCO_3_^-^ and CO_3_^2-^ significantly impacted phosphate removal, mainly due to their alkalinity, which raises the solution pH (Yang et al., 2018). When the pH exceeds 10.0, Fe-B’s phosphate adsorption efficiency decreases significantly. Additionally, previous reports indicated that HCO_3_^-^ and CO_3_^2-^, as anions, can generate electrostatic repulsion with phosphate ions, further reducing adsorption (Qu et al., 2020).

**Figure 1D** illustrates a gradual decline in the adsorption capacity of Fe-B when eluted with NaOH solution. Initially, after the first desorption, the capacity decreased from 2.05 mg·g^-1^ to 1.55 mg·g^-1^, indicating that Fe-B retained considerable adsorption ability. However, by the fifth desorption, the capacity had diminished to 24%, indicating a significant reduction in recyclability. This decline in phosphate adsorption capacity may be attributed to several factors. First, prolonged exposure to high-concentration NaOH solution could elute iron oxides from Fe-B, diminishing its adsorption efficiency. Second, when Fe-B reaches adsorption saturation, the desorption solution might not fully remove the phosphate from both the surface and internal structure of Fe-B (Rahman et al., 2022; Zhao et al., 2019). As a result, phosphate may occupy active adsorption sites and form precipitates that block Fe-B’s pore structure. This blockage hinders the entry of additional phosphate into the porous structure, preventing binding to internal adsorption sites and thereby reducing the overall adsorption performance of Fe-B (Wu et al., 2020).

As the reaction time progresses, phosphate adsorption by Fe-B is initially rapid, but the rate gradually decreases until equilibrium is achieved (**Figure 1E**). To analyze the adsorption kinetics, the data were fitted to both pseudo-first-order and pseudo-second-order kinetic models. The correlation coefficient (R^2^) for the pseudo-second-order model was 0.9757, which is higher than that for the pseudo-first-order model (**Supplementary Table S1**). This suggests that the adsorption of phosphate by Fe-B aligns more closely with the pseudo-second-order kinetic model, indicating that the primary adsorption mechanism is chemical (Ou et al., 2023). **Figure 1F** demonstrates that the equilibrium adsorption capacity of Fe-B increases with rising initial phosphate concentrations, eventually plateauing. This trend correlates with the ratio of available adsorption sites on the Fe-B surface. Phosphate adsorption data for Fe-B were analyzed using the Langmuir and Freundlich models. As indicated by the fitting parameters in **Supplementary Table S2**, both models yielded correlation coefficients (R^2^) > 0.9, with the Langmuir model exhibiting a higher R^2^. This suggests that the Langmuir model more accurately describes the phosphate adsorption by Fe-B, suggesting a predominantly monolayer adsorption process (Kang et al., 2021).

### 3.2 Characterization of Fe-B

**Figure 2:**
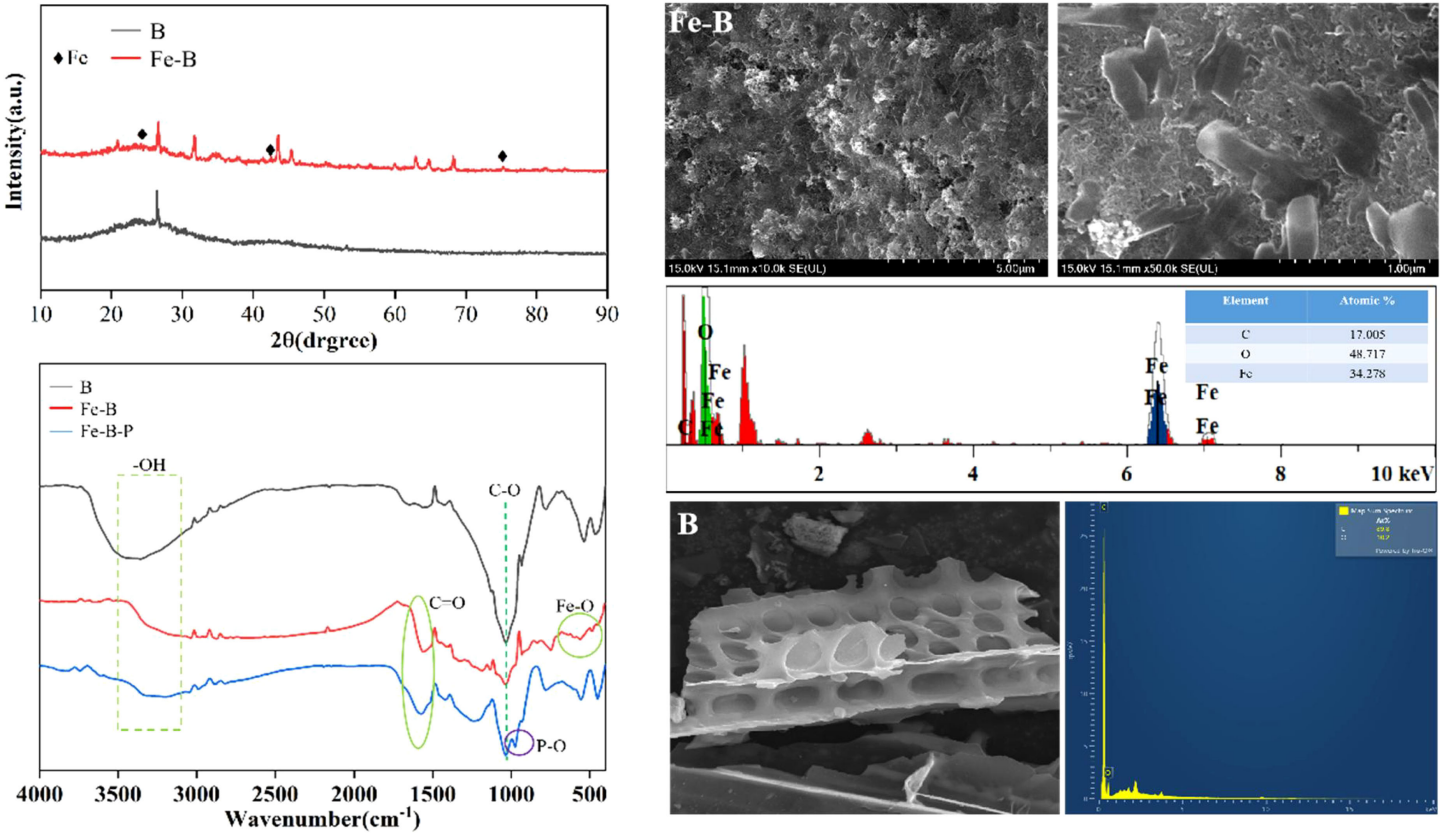
Characterization of FeB and P-loaded FeB.

The unmodified biochar surface is smooth, with a distinct porous structure and minimal impurities or particulate matter (**Figure 2**). In contrast, the Fe-modified biochar surface exhibits a small amount of particulate load while retaining its porous structure (Zhang et al., 2023). The EDS spectra reveal that the original biochar consists only of C and O. Following modification, the biochar iron content increases to over 34%. The functional group peaks on both the Fe-B and original biochar surfaces are similar in the 3300–3500 cm^-^¹ range, though they differ significantly in intensity. This variation may result from iron hydroxide covering some functional groups or the absorption of hydroxyl groups due to the hydrolysis of FeCl_3_ (Kong et al., 2023). The absorption peaks of Fe-modified biochar in the 500–700 cm^-^¹ range show significant differences in both intensity and type before and after adsorption, indicating that phosphate adsorption by Fe-modified biochar is a chemical process (Ajmal et al., 2020). After phosphate adsorption, the stretching vibration band at 974 cm^-^¹ in the Fe-modified biochar corresponds to the P-O single bond. Additionally, new peaks at 1550 cm^-^¹ in both Fe-B and adsorbed Fe-B are attributed to the stretching vibration of C=O, indicating an increased presence of oxygen-containing functional groups and enhanced adsorption capacity.

### 3.3 Yield and phosphorus fertilizer utilization efficiency

**Figure 3:**
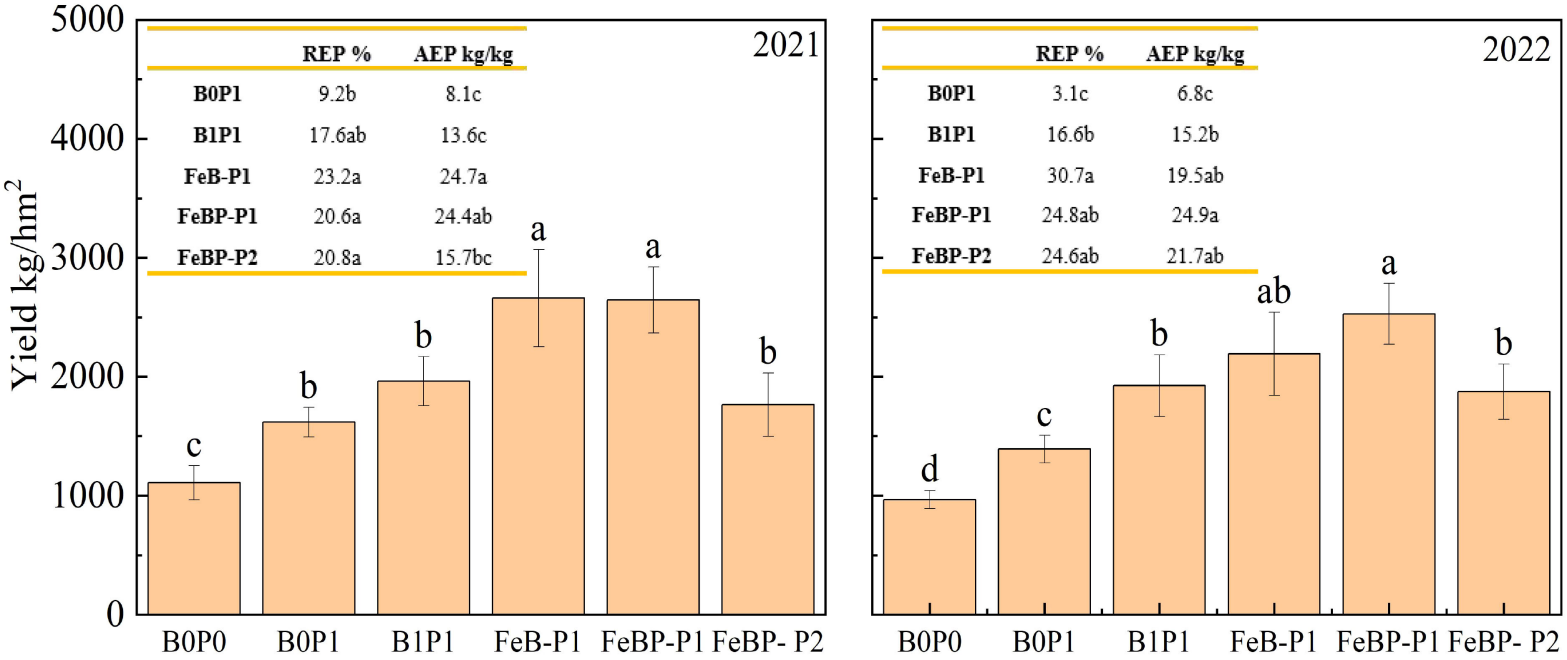
Yield and utilization efficiency of phosphate fertilizer. REP, Recovery efficiency of phosphorus; AEP, Agronomic efficiency of phosphorus.

The peanut yield under the FeB-P1 treatment increased by 64.5% and 35.6% compared to the B0P1 and B1P1 treatments in 2021, respectively (**Figure 3**). There was no significant difference in yield, recovery efficiency of phosphate fertilizer (REP), and agronomic efficiency of phosphate fertilizer (AEP) between the FeB-P1 and FeBP-P1 treatments. This shows that increasing the amount of phosphate fertilizer cannot increase the yield, and reduce the utilization efficiency of phosphate fertilizer. This is consistent with most studies, phosphorus reduction does not necessarily reduce production, but improves the efficiency of phosphorus fertilizer utilization (Rakotoson et al., 2022; Cao et al., 2021). When considering the two-year comprehensive data, in 2021, there was no difference in yield between FeBP-P1 treatment and FeBP treatment, but both were higher than FeBP-P2. In 2022, the difference between FeBP-P1 and FeBP treatment was not significant, which may be due to the slow release of phosphorus by iron-modified biochar. Amin and Mihoub (2021) also found in their studies that Sulfur-Enriched biochar acted as a slow-release phosphate fertilizer, which can increase the available phosphorus content and P associated with calcium fractions in soil. The yield of FeBP-P2 decreased by 33.3% compared with that of FeBP-P1, but there was no significant difference in REP between FEBP-P2 and FEBP-P1.While the FeBP-P2 treatment resulted in a 9.1% yield increase over B0P1, this difference was not statistically significant. Notably, the REP under FeBP-P2 was 126.1% higher than under B0P1 (P < 0.05). No significant difference in REP was observed between the FeBP-P2 and B1P1 treatments, suggesting that the application of Fe-BP under reduced phosphorus conditions maintained yield while enhancing phosphorus fertilizer utilization efficiency.

### 3.4 Available phosphorus in soil profile

**Figure 4:**
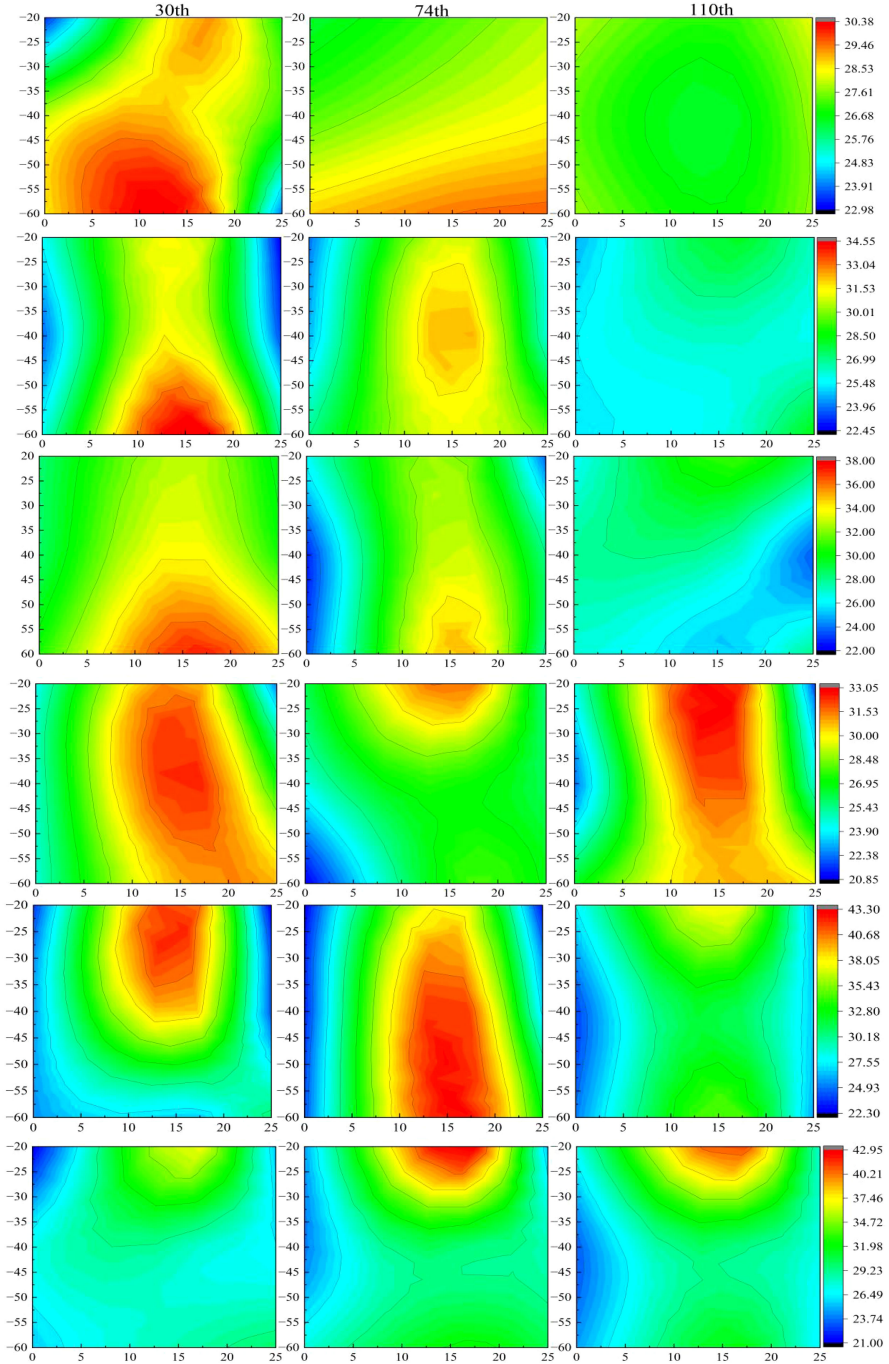
Dynamic changes of spatial distribution of soil available phosphorus during growth period.30th, 74th and 110th represent the number of days after seedling.The six images from top to bottom are processed as B0P0, B1P1, B0P0, FeB-P1, FeBP-P1, FeBP-P2. B0P1 represents no biochar and no phosphorus fertilizer, B0P1 represents no biochar and conventional phosphate fertilizer, B1P1 represents biochar and conventional phosphate fertilizer, FeB-P1 represents iron-modified biochar and conventional phosphate fertilizer, FeBP-P1 represents phosphorus-loaded Fe-B and conventional phosphate fertilizer, FeBP-P2 represents phosphorus-loaded Fe-B and two third conventional phosphate fertilizer.

The surface layer of the B0P0 treatment exhibited extremely low available phosphorus levels (**Figure 4**). Throughout the growing season, factors such as rainfall and irrigation led to the leaching of nearly all the original surface phosphorus from the soil (Aulakh et al., 2007). In the B0P1 and B1P1 treatments, the distribution and variation of available phosphorus were similar, suggesting that the original biochar had a poor capacity for phosphorus adsorption. This is similar to the research of some scholars, who believe that the phosphorus in biochar is stable in structure and difficult to dissolve, especially the biochar with low phosphorus content cannot increase the phosphorus content in soil (Qian et al., 2013). It is also believed that the adsorption of phosphate by biochar will essentially compete with plants and reduce the concentration of phosphorus in soil solution (Zhang et al., 2024). In contrast, the FeB-P1 treatment demonstrated superior phosphorus adsorption, with only slight leaching of available phosphorus at the end of the growing season. A decrease in available phosphorus content during the mid-growing season may be attributed to the presence of additional adsorption sites on FeB, which adsorbed phosphate in the soil (Zhang et al., 2016). This phenomenon was not observed with phosphate-loaded iron-modified biochar, as its adsorption sites were already occupied. When phosphate-loaded iron-modified biochar was applied under reduced phosphorus conditions, the available phosphorus remained concentrated near the root zone, with stable but lower levels than in other treatments, and showed no significant dynamic changes throughout the season. This stability may result from the combined effect of reduced phosphorus fertilizer and the slow-release function of phosphate-loaded iron-modified biochar (Talboys et al., 2016). It has also been reported that the modified biochar makes the soil effective phosphorus release period longer, The rate of P diffusion in biochar-amended soils was lower than the unamended soil (Mihoub et al., 2022). The phosphorus fertilizer utilization efficiency in this treatment indicated a stable and continuous release of available phosphorus, reduced phosphorus loss, and higher utilization efficiency.

### 3.5 Roots

It can be seen from the **Table 1** that not applying phosphorus fertilizer significantly inhibits the development of peanut root. The root surface area of B0P1 treatment was 33.25% and 39.38% higher than that of B0P0 treatment, respectively, at flower-pegging stage and pod setting stage in 2021. By 2022, this situation became more obvious, with the root surface area of B0P1 treatment being 96.21% higher than that of B0P0 treatment. Péret et al. (2011) has found that soil phosphorus can stimulate the development of lateral roots and root hairs, especially for the seedling stage of crops. Sufficient phosphorus can accelerate the early development of the root system, increase the root surface area, while long-term phosphorus deficiency will limit root development, and the longer the deficiency, the more detrimental it will be to the root system (Liu et al., 2023), which is consistent with the results of this study. This study found that in the early growth period, biochar had no significant effect on the development of peanut root. There was no significant difference in root surface area between B1P1 and B0P1 treatments from the 2021 flower-pegging stage to the 2022 flower-pegging stage, but the root surface area of B1P1 treatment was significantly higher than that of B0P1 treatment by 25.83% in the pod setting stage of 2022, which may be due to the phosphorus release effect of biochar (Gwenzi et al., 2018; El-Naggar et al., 2019). This delayed release effect was more obvious in the FeBP P1 treatment, with the root surface area of FeBP P1 treatment being 39.52% higher than that of B0P1 treatment (P<0.05) and 10.88% higher than that of B1P1 treatment (P>0.05) at pod setting stage in 2022. There have been studies showing that phosphorus-release fertilizers can help crop root development and improve root vitality (Li G. et al., 2022; Everaert et al., 2016). The root length, root surface area, and root volume of the FeBP P1 treatment were all higher than those of the FeBP P2 treatment at pod-setting stage in 2021. However, there was no significant difference in these indicators between the two treatments at pod setting stage in 2022, which suggests that even under conditions of reduced phosphorus fertilizer application, the use of slow-release phosphorus fertilizers can promote root development, but with a certain lag. Therefore, the phosphorus release rate of FeBP as a slow-release fertilizer still needs to be further developed.

**Table 1:** ANOVA output of different treatments on root length, root surface area and root volume.

Stage	Treatments	Total root Length (cm)		Root Surface Area (cm^2^)		Root Volume (cm^3^)	
		2021	2022	2021	2022	2021	2022
Flower-pegging stage	B0P0	362.19b	403.19b	47.86c	42.78c	0.74c	0.63b
	B0P1	450.11ab	496.01b	63.77b	83.94b	1.03b	1.01a
	B1P1	479.02a	500.53b	67.64b	99.10ab	1.07b	1.00a
	FeB P1	536.51a	686.87a	83.29a	112.91a	1.23a	1.10a
	FeBP P1	532.47a	628.02a	86.35a	113.29a	1.25a	1.04a
	FeBP P2	493.62a	493.36b	67.41b	92.58ab	1.07b	1.05
Pod setting stage	B0P0	450.95d	657.56c	85.39d	89.23c	0.92c	1.05b
	B0P1	588.91c	1291.39b	119.02abc	108.34bc	1.23b	1.24ab
	B1P1	639.99bc	1335.34b	115.31bc	136.32a	1.29b	1.28a
	FeB P1	755.60ab	1525.73ab	134.07ab	127.54ab	1.66a	1.02ab
	FeBP P1	846.13a	1635.40a	137.13a	151.16a	1.32b	1.23ab
	FeBP P2	662.65bc	1429.15ab	108.83c	134.86a	1.13bc	1.20ab

For interaction effect, mean data (n = 3) with different letters are significantly different at P < 0.05.

## 4 Conclusions

In this study, the phosphate adsorption by iron-modified biochar (Fe-B) was characterized as chemisorption and monolayer adsorption. Within a specific pH range, Fe-B demonstrated effective phosphate adsorption and could mitigate interference from competing ions. Post-adsorption, the iron-modified biochar showed potential as a phosphorus slow-release fertilizer (Fe-BP). When phosphate fertilizer input was reduced, Fe-BP maintained the available phosphorus content in the peanut rooting zone, thereby not only sustaining peanut yield but also enhancing phosphate fertilizer utilization efficiency. Therefore, it is more economical to apply FeBP in the case of reducing the input of phosphate fertilizer. However, this new type of phosphorus-release fertilizer has a slower release rate, so improving its release efficiency is the focus of the next stage of research.

## Data Availability

The original contributions presented in the study are included in the article/**Supplementary Material**. Further inquiries can be directed to the corresponding author/s.
