# Supplementary material for: Reduce the application of phosphorus fertilizer in peanut fields and improve its efficiency by using iron modified biochar to adsorb phosphorus recovery products
Source: Front Plant Sci. 2024 Dec 17;15:1515584. doi: 10.3389/fpls.2024.1515584 (PMC11687223; doi:10.3389/fpls.2024.1515584)
Supplement: Supplementary file 1 [file DataSheet1.docx]

**Supplemental information**

Table S1 Soil conditions

| Type | pH , | Bulk density, | Field capacity | Total nitrogen | Available potassium | Available phosphorus | Organic matter |
| --- | --- | --- | --- | --- | --- | --- | --- |
| Sandy loam | 6.12 | 1.69g cm^-3^ | 15.99% | 0.74g kg^-1^ | 19.1mg kg^-1^ | 1.7 mg kg^-1^ | 1.7g kg^-1^ |

Table S2. Fitting parameters of the kinetic model of phosphate adsorption by iron-modified biochar

| Pseudo-first-order model | | | Pseudo-second-order model | | |
| --- | --- | --- | --- | --- | --- |
| $q_{e}$ | $k_{1}$ | R^2^ | $q_{e}$ | $k_{1}$ | R^2^ |
| 3.89±0.1 | 0.044±0.0004 | 0.9718 | 4.11±0.0.11 | 0.015±0.002 | 0.9757 |

Table S3. The fitting parameters of the isothermal adsorption model for phosphate adsorption by iron-modified biochar

| Langmuir | | | Freundlich | | |
| --- | --- | --- | --- | --- | --- |
| $\text{q}_{\text{m}}$ | $k_{l}$ | R^2^ | $k_{f}$ | n | R^2^ |
| 11.73±0.72 | 0.0857±0.012 | 0.9863 | 0.69±0.43 | -0.6243±0.026 | 0.9351 |


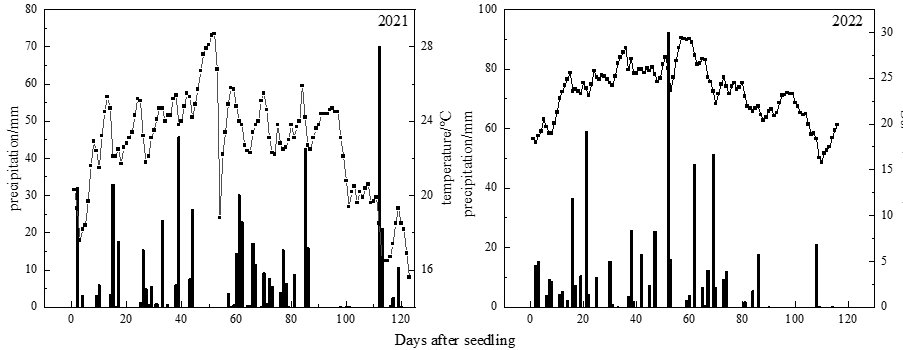


Fig. S1. Daily precipitation and temperature during the 2021 and 2022 growing seasons.
